# Metabolic engineering of *Saccharomyces cerevisiae* for the biosynthesis of a fungal pigment from the phytopathogenic fungus *Cladosporium phlei*

**DOI:** 10.1186/s13036-024-00429-0

**Published:** 2024-05-13

**Authors:** Yeji Gwon, Kum-Kang So, Jeesun Chun, Dae-Hyuk Kim

**Affiliations:** 1 Department of Bioactive Material Sciences, Jeonbuk National University, Jeonju, 54896 Republic of Korea; 2 Institute for Molecular Biology and Genetics, Jeonbuk National University, Jeonju, 54896 Republic of Korea; 3 Department of Molecular Biology, Jeonbuk National University, Jeonju, 54896 Republic of Korea

**Keywords:** Phleichrome, *Cladosporium phlei*, *Saccharomyces cerevisiae*, Polyketide synthase

## Abstract

**Background:**

*Cladosporium phlei* is a phytopathogenic fungus that produces a pigment called phleichrome. This fungal perylenequinone plays an important role in the production of a photosensitizer that is a necessary component of photodynamic therapy. We applied synthetic biology to produce phleichrome using *Saccharomyces cerevisiae.*

**Results:**

The gene *Cppks1*, which encodes a non-reducing polyketide synthase (NR-PKS) responsible for the biosynthesis of phleichrome in *C. phlei*, was cloned into a yeast episomal vector and used to transform *S. cerevisiae*. In addition, a gene encoding a phosphopantetheinyl transferase (PPTase) of *Aspergillus nidulans* was cloned into a yeast integrative vector and also introduced into *S. cerevisiae* for the enzymatic activation of the protein product of *Cppks1*. Co-transformed yeasts were screened on a leucine/uracil-deficient selective medium and the presence of both integrative as well as episomal recombinant plasmids in the yeast were confirmed by colony PCR. The episomal vector for *Cppks1* expression was so dramatically unstable during cultivation that most cells lost their episomal vector rapidly in nonselective media. This loss was also observed to a less degree in selective media. This data strongly suggests that the presence of the *Cppks1* gene exerts a significant detrimental effect on the growth of transformed yeast cells and that selection pressure is required to maintain the *Cppks1*-expressing vector. The co-transformants on the selective medium showed the distinctive changes in pigmentation after a period of prolonged cultivation at 20 °C and 25 °C, but not at 30 °C. Furthermore, thin layer chromatography (TLC) revealed the presence of a spot corresponding with the purified phleichrome in the extract from the cells of the co-transformants. Liquid chromatography (LC/MS/MS) verified that the newly expressed pigment was indeed phleichrome.

**Conclusion:**

Our results indicate that metabolic engineering by multiple gene expression is possible and capable of producing fungal pigment phleichrome in *S. cerevisiae*. This result adds to our understanding of the characteristics of fungal PKS genes, which exhibit complex structures and diverse biological activities.

## Background

**Fig. 1:**
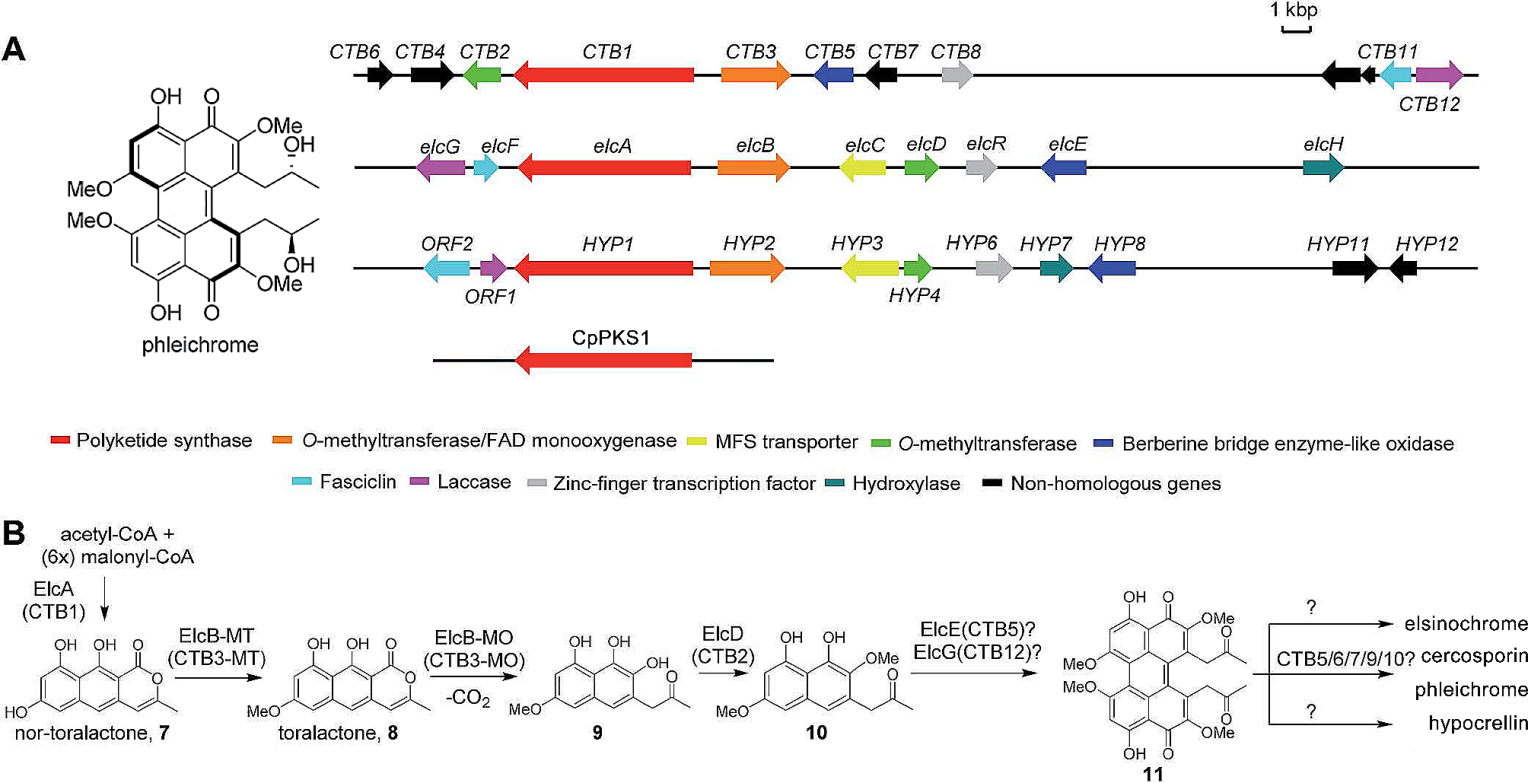
Metabolic pathways for the synthesis of fungal perylenequinone adapted from Hu et al., 2019 **(A)**. *Cppks1* genes, responsible for phleichrome biosynthesis, are indicates as red. The chemical structure of phleichrome adapted from Seto et al., 2005 **(B)**

The fungus *Cladosporium phlei* (C. T. Gregory) de Vries, a causal agent of leaf spot disease of timothy (*Phleum pratense*) (known as a purple eyespot), produces phleichrome, a deep red pigment [1, 2]. Phleichrome belongs to a group of fungal perylenequinones and is identified as a 1,12-bis-(2-hydoxypropyl)-2,6,7,11-tetramethoxy-4,9-dihydroxyperylene-3,10-quinone harboring 4,9-dihydroxyperylene-3,10-quinone chromophore [2] (Fig. 1). Although phleichrome acts as a virulence factor in the most common foliar disease of timothy, the characteristic symptoms of which include circular purple and then brown spots on the leaves with white to grayish-fawn centers [2–4] and an antimicrobial factor to neighboring microorganism, it is also being studied for its therapeutic potential as a photosentitizer for photodynamic therapy (PDT) [5, 6].

PDT, which requires both a selective photosensitizer and a spectrum-matching light source, is an innovative and promising means of treating cancer, other obstinate diseases, and antibiotic-resistant bacterial strains [7, 8]. During PDT treatment a photosensitizer is activated by absorption of a certain wavelength from a light-source, which then generates short-lived toxic species that treat disease and pathogens. Our previous studies revealed the photodynamic activity of phleichrome when illuminated, as it converts oxygen into reactive oxygen species (ROS) [5, 6].

Recent studies on the biosynthesis of fungal perylenequinones have shown that most fungal perylenequinones including a close analog of phleichrome, are synthesized via the polyketide pathway [4, 9]. Phleichrome is synthesized in this manner as well [5, 10]. Polyketide synthases (PKSs) are multimeric enzymes functioning analogously to fatty acid synthase, in which carboxylic acid units responsible for biosynthesis of all fungal polyketides are joined in a stepwise fashion [11]. Multiple PKS genes are present for encoding multiple PKSs in the fungal genome. Our ordered fosmid library screening [10] and genome sequencing approaches [12] revealed at minimum of eight PKS genes in the *C. phlei* genome. We have also shown that the *Cppks1* gene, encoding a non-reducing PKS, is responsible for the biosynthesis of phleichrome [5]. Although a few biosynthetic gene clusters for closely related fungal perylenequinones, such as cercosporin, elsinochrome, and hypocrellin have been described [13–15], the gene cluster for the biosynthesis of phleichrome and its corresponding metabolites needs to be characterized (Fig. 1).

Baker’s yeast *Saccharomyces cerevisiae* has long been used as a model experimental system for basic as well as applied research for its huge amount of genetic, molecular, cellular, and engineering information [16–23]. In addition, *S. cerevisiae* is a generally recognized as safe (GRAS) organism, which renders its expressed products and associated processing more entrusted than other expression systems. Within the area of biotechnology, it is the most popular eukaryotic host for the production of foreign gene proteins and shows tremendous potential as part of metabolic engineering systems capable of producing heterologous compounds that require a non-native biosynthetic pathway.

*S. cerevisiae* has been used for the heterologous expression of the 6-methylsalicylic acid (6-MSA) synthase responsible for 6-MSA biosynthesis, which is the first fungal PKS cloned and characterized [24–26]. The heterologous biosynthesis of polyketides in *S. cerevisiae* requires the covalent attachment of 4’-phosphopantetheine moiety of CoA to the acyl carrier protein (ACP) domain of PKS for its own activation by 4’-phosphopantetheinyl transferases (PPTases) [24, 27]. Therefore, we set out to metabolically engineer *S. cerevisiae* for heterologous biosynthesis of the fungal pigment phleichrome. We achieved this by co-expressing a phleichrome biosynthetic PKS gene from *C. phlei* (*Cppks1*) and PPTase gene from *Aspergillus nidulans* (*npgA*) in our bred *S. cerevisiae* 2805-a7 strain.

## Methods

### Strains and culture conditions

Plasmids were maintained and propagated in *E. coli* Top10 or DH5α consistent with Sambrook et al., 2001 [28]. *S. cerevisiae* 2805-a7 (* pep4::HIS3 leu2-3 ura3-52*) was used as the recipient cell for transformation [29]. The *S. cerevisiae* culture was maintained in a YEPD medium (1% yeast extract, 2% peptone, and 2% dextrose) while uracil-deficient (*ura*^*−*^) selection medium [0.67% yeast nitrogen base without amino acids (Sigma-Aldrich), 1.92 g/L yeast synthetic drop-out media supplements w/o uracil (Sigma-Aldrich), 2% glucose, and 2% agar], leucine-deficient (*leu*^*−*^) selection medium [0.67% yeast nitrogen base without amino acids (Sigma-Aldrich), 1.92 g/L yeast synthetic drop-out media supplements w/o leucine (Sigma-Aldrich), 2% glucose, and 2% agar], and double-deficient (*leu*^*−*^/*ura*^*−*^) selection medium [0.67% yeast nitrogen base without amino acids (Sigma-Aldrich), 1.46 g/L yeast synthetic drop-out media supplements w/o uracil, leucine, and tryptophan, 76 mg/L tryptophan, 2% glucose, and 2% agar] were used to screen transformants at 30 °C. For liquid cultures, a healthy transformant colony was inoculated into 5 mL selection medium and incubated for 48 h at 30 °C with continuous agitation (200 rpm). Subsequently, 250 uL of the primary inoculum was inoculated into 5 mL of YEPD medium and incubated for 16 h at 30 °C with continuous agitation (200 rpm). This 5 mL culture was transferred into a 300-mL Erlenmeyer flask containing 40 mL of selection medium at 20 °C, 25 °C, and 30 °C with continuous agitation (200 rpm), after which the cells were harvested and examined for phleichrome expression [30–32].

### Constructions of expression vectors

**Fig. 2:**
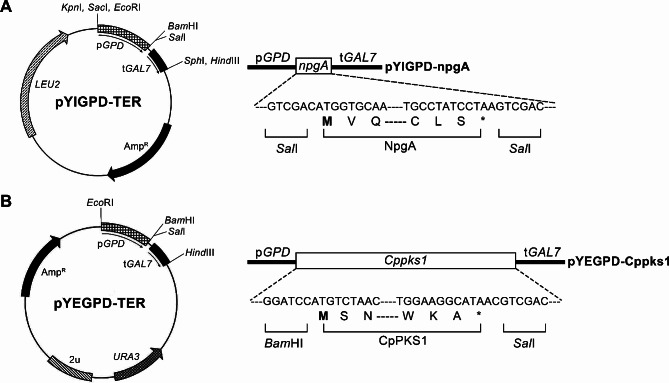
Restriction map of transforming vectors for *npgA* (**A**) and *Cppks1* (**B**)

The encoding genes for *npgA* (GenBank No. AF198117.1) and *Cppks1* (GenBank No. JX129223.1) were cloned into a pGEM®-T easy vector for further manipulation and the clones were verified by DNA sequencing. For expression vectors, each construct was prepared using the PCR method to contain appropriate restriction enzyme sites at the 5’ and 3’ termini. The constructs were subsequently digested with appropriate restriction enzymes and cloned into the yeast expression vectors between the glyceraldehyde-3-phosphate dehydrogenase (*GPD*) promoter and the galactose-1-phosphate uridyl transferase (*GAL7*) terminator [33, 34]. The *npgA* was cloned into the *Sal*I site in a low-copy integrative pYIGPD-TER vector and the *Cppks1* was cloned into the *Bam*HI/*Sal*I sites in a high-copy episomal pYEGPD-TER vector. The same promoter was used for both vectors to minimize any discrepancies attributable to differences in promoter strength. The expression vectors for *npgA* and *Cppks1* were respectively named pYIPGPD-npgA and pYEGPD-Cppks1. The restriction maps of both the integrative and episomal recombinant plasmids are shown in Fig. 2.

The *S. cerevisiae* strain was transformed as previously reported [30–32, 35, 36]. The transformed cells were selected on the selection medium, and the presence of the transforming plasmid was confirmed by colony PCR or back-transformation of *E. coli* with DNA prepared from the putative transformants [31].

The stability of the introduced plasmids in yeast was measured using appropriate selection media according to a previously described procedure [37]. Vectors for yeast transformation, recombinant yeast strains, and primers for construction of these vectors are indicated in Tables 1 and 2, and Table 3, respectively.

**Table 1:** Plasmids employed in this study

Plasmid	Description	Reference
pYEGPD-TER	YEp352 with the *GPD* promoter-*GAL7* terminator	[42]
pYIGPD-TER	YIplac128 with the *GPD* promoter-*GAL7* terminator	[33]
pYEGPD-Cppks1	*Cppks1* in pYEGPD-TER plasmid	This study
pYIGPD-npgA	*npgA* in pYIGPD-TER plasmid	This study

**Table 2:** *S. cerevisiae* strains employed in this study

Strain	Genotype	Parent strain	Reference
2805-a7	*pep4*::*HIS3 leu2-3 ura3-52*	2805	[42]
TYI	*pep4*::*HIS3 leu2-3 ura3-52::LEU2*	2805-a7	This study
TYE	*pep4*::*HIS3 leu2-3 ura3-52::URA3*	2805-a7	This study
TYINPGA	*pep4*::*HIS3 leu2-3 ura3-52::LEU2-NPGA*	2805-a7	This study
TYECPPKS1	*pep4*::*HIS3 leu2-3 ura3-52::URA3-CPPKS1*	2805-a7	This study
TYCO	*pep4*::*HIS3 leu2-3::LEU2-NPGA ura3-52::URA3-CPPKS1*	TYINPGA	This study

**Table 3:** List of primers employed in this study

Primer	Sequence (5’ to 3’)
SalI-NpgA-F	GC*GTCGA*CATGGTGCAAGACACATCAAGCGCA
SalI-NpgA-R	GC*GTCGA*CTTAGGATAGGCAATTACACACCCCAGTC
BamHI-Cppks1-F	GCT*GGATCC*ATGTCTAACGTTCTCCTC
SgrDI-Cppks1-R	CTA*CGTCGACG*TTATGCCTTCCAGTCGAC

Italicized indicate restriction enzyme sites

### Analysis of the transformants

Recombinant yeast was analyzed using thin-layer chromatography (TLC) followed by liquid chromatography (LC/MS/MS). Briefly, ethyl acetate (EtOAc) was used to extract phleichrome from the yeast cells [38, 39]. The yeast cells were broken with beads and EtOAc using FastPrep-24™ 5G (MP biomedicals). The broken cells with EtOAc were then gently agitated for 5 h at 1,500 rpm using CUTE MIXER CM-1000 (EYELA). The crude extract was then resolved using TLC on silica gel with a resolving solution (CH_2_Cl_2_/MeOH = 19:1, v/v) and purified phleichrome as a control [5]. The band with mobility most similar to that of the purified phleichrome was scraped out from the silica gel and dissolved in methanol, and the presence of phleichrome was further analyzed by LC/MS/MS [5].

### Northern blot analysis and real-time RTPCR

Transformants were grown in YEPD medium, and total yeast RNA was extracted as previously described [37]. The RNA concentration was determined using a Multiskan GO (Thermo Fisher Scientific Inc.). To evaluate the expression levels of the target genes, northern blot analysis and quantitative real-time RT-PCR were performed as previously described [40].

### LC/MS/MS analysis

Unbiased metabolomics analysis was performed as previously described using an ultra-performance liquid chromatography (UPLC) system (Waters, Milford, USA) [41]. The scan range in MS and MS/MS modes included 50–1200 m/z. Data acquisition and analysis were controlled by Waters UNIFI V1.71 software. The metabolic profile of the TLC-purified pigment from the transformed *S. cerevisiae* was compared with that of the purified phleichrome from *C. phlei* obtained in our previous studies [5, 38, 39].

## Results and discussion

### Co-expression of ***npgA*** and ***Cppks1*** in recombinant yeast

**Fig. 3:**
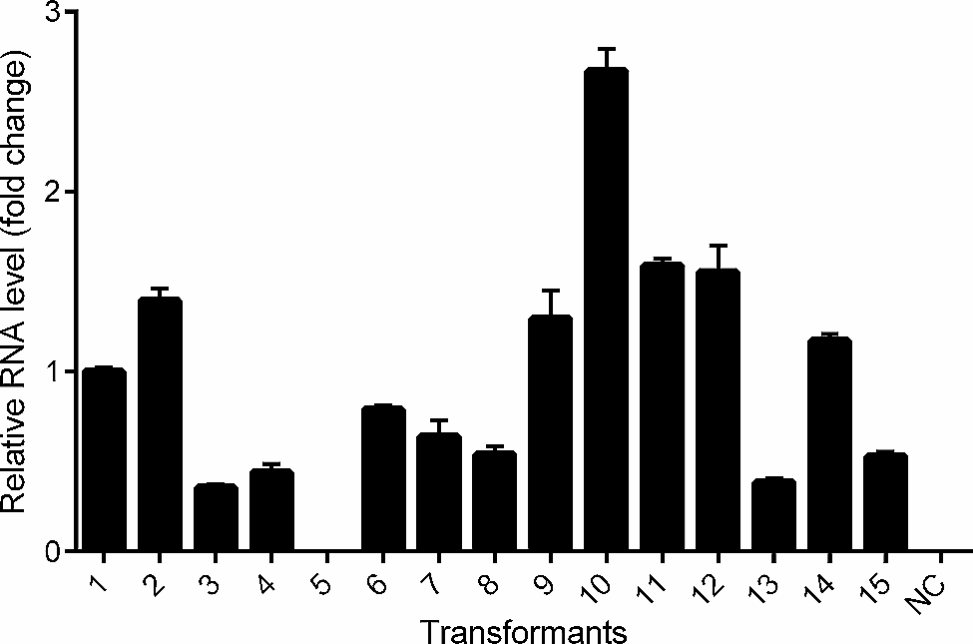
Quantitative real-time RT-PCR (qRT-PCR) analysis of the *npgA* gene transformants grown at 30 ºC for 3 days. Transcript levels of *npgA* are shown as the fold changes relative to that of the *GPD* transcript. Error bars indicate standard deviation based on three independent measurements

The pYIGPD-npgA plasmid was constructed to express the PPTase-encoding *npgA* gene using a low-copy integrative vector (pYIGPD-TER) as described in the Methods section (Fig. 2). The resulting plasmid was used to transform the *S. cerevisiae* 2805-a7 strain. Over 15 colonies were randomly selected on a *leu*^-^ selective medium and then examined for the presence of pYIGPD-npgA via PCR amplification of the integrated *npgA* gene from the extracted chromosomal DNA. Quantitative real-time RT-PCR was conducted to verify the expression of the cloned *npgA* gene. Two transformants (TYINPGA-10 and − 11), showing high accumulation of the *npgA* transcript from the 3-day-old culture, were selected for further transformation of the *Cppks1* gene (Fig. 3).

**Fig. 4:**
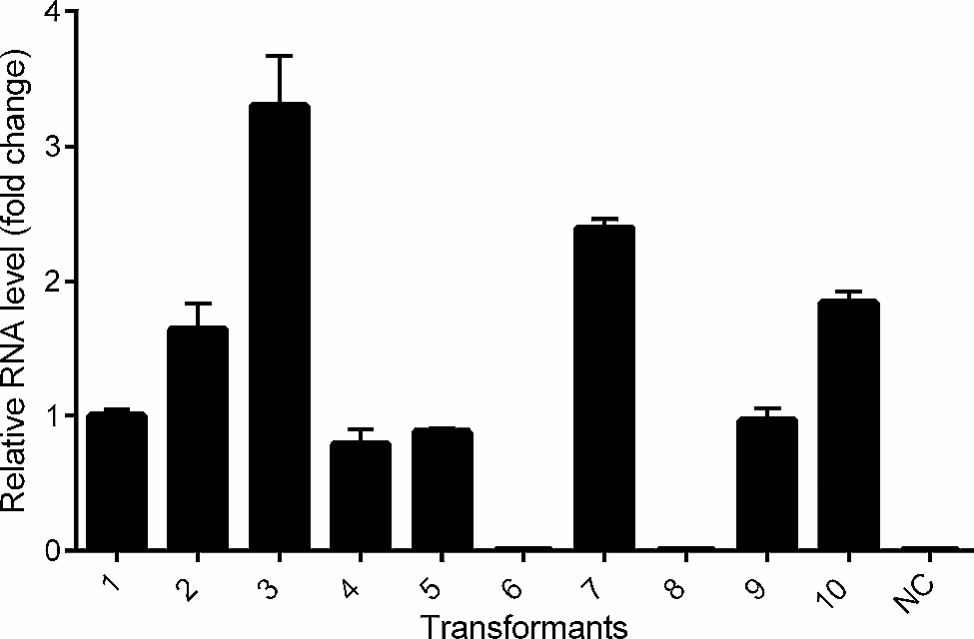
Quantitative real-time RT-PCR (qRT-PCR) analysis of co-transformants cultured at 30 ºC for 3 days. Transcript levels of the *Cppks1* gene are represented as the fold changes relative to that of the *GPD* transcript. Error bars indicate standard deviation based on three independent measurements. Co-transformants from TYINPGA-10, represented by columns 1,2,3,4,5, and 7, are referred to as TYCO-1, -2, -3, -4, -5, and − 6, respectively. Co-transformants from TYINPGA-11, represented by columns 9 and 10, are referred to as TYCO-7 and − 8, respectively

To produce phleichrome via the co-expression of the *npgA* and *Cppks1* genes, a high-copy episomal vector, pYEGPD-TER, was engineered to express the *Cppks1* gene for PKS and the resulting recombinant vector pYEGPD-Cppks1 was used to transform two selected transformants (TYINPGA-10 and − 11) expressing the *npgA* gene. Over 10 colonies for each transformant were selected randomly on an *ura*^−^ medium and then examined for the presence of the pYEGPD-Cppks1 vector via colony PCR amplification of the *Cppks1* gene. The pYEGPD-Cppks1 vector was then rescued and verified by restriction enzyme analysis. Quantitative real-time RT-PCR was performed to select the co-transformant with the highest *Cppks1* gene expression levels. A total of eight transformants, i.e., six from TYINPGA-10 (TYCO-1, -2, -3, -4, -5, and − 6) and two from TYINPGA-11 (TYCO-7 and − 8), were selected for their considerable expression and used for further analysis of phleichrome production (Fig. 4).

### Plasmid stability and cell growth of recombinant strains

Stability of the episomal recombinant plasmid and cell growth were assessed by counting the number of cells with the hemocytometer followed by comparing colony forming units (CFUs) on nonselective YEPD with selective *ura*^*−*^ media. The co-transformant TYCO-3 from TYINPGA-10 and co-transformant TYCO-7 from TYINPGA-11 were examined for plasmid stability. Plasmid stability of the transformants grown in the YEPD broth for 3 days was measured by plating approximately 100 cells (estimated by hemocytometer) on a *ura*^*−*^ selective plate and a nonselective YEPD plate at 30 °C. Compared to those of nonselective media, less than 5% of the CFUs were observed from the selective media, which suggests that more than 95% of the cells lost plasmid after the 3-day incubation (Table 4). No significant differences in CFUs were observed between nonselective YEPD plate and *leu*^*−*^ selective plate, indicating that cells grown in YEPD broth for our analysis were indeed our engineered transformants losing episomal plasmid. This instability ratio was significantly higher than those of the control mock transformants (transformed with an episomal vector without the *Cppks1* gene) in which more than 80% of the plated cells appeared to carry the plasmid 3 days after liquid culture. Such high plasmid instability was not previously observed in our prior studies of similar episomal plasmids [42, 43]. Interestingly, although it was not as high as for YEPD media, significantly fewer CFUs (≤ 35%) from the cells grown in *ura*^*−*^ selective broth were also observed on selective media, i.e., more than 65% of plated cells were unable to grow to form distinct colonies on a *ura*^*−*^ selective plate. This suggests that many cells that survived during cultivation in *ura*^*−*^ selective broth were unable to multiply to form colonies on the *ura*^*−*^ selective plate. Although further studies investigating the effect of plasmid size on instability need conducting, the remarkable instability of the episomal recombinant vector for the expression of the *Cppks1* gene of co-transformed cells suggests that both the intrinsic instability of episomal plasmid for the expression of the *Cppks1* gene as well as the biosynthesis of metabolite phleichrome contributed to plasmid instability. Accumulation of phleichrome appears to have been detrimental to the growth of fungal host *S. cerevisiae*, which is consistent with phleichrome’s behavior as an antimicrobial factor [44]. Therefore, we examined the cell growth of co-transformants.

**Table 4:** CFUs of recombinant strains on YEPD and *ura*^*-*^ selective media

Strain	CFUs/plate ^a, b^	
	YEPD	ura^−^
TYE		
YEPD	77.3 ± 4.0^c^	62.66 ± 10.1
*ura*^*−*^	32.3 ± 7.4	28.0 ± 1.7
TYCO-3		
YEPD	109.0 ± 12.9	3.0 ± 2.0
*ura*^*−*^	80.3 ± 11.2	17.3 ± 6.7
TYCO-7		
YEPD	105.0 ± 13.5	2.7 ± 1.2
*ura*^*−*^	71.0 ± 10.6	25.7 ± 12.0

^a^Colony-forming units per plate

^b^For each strain, 1 × 10^2^cells were spread on the media

^c^Values are shown as the means ± standard deviation

**Fig. 5:**
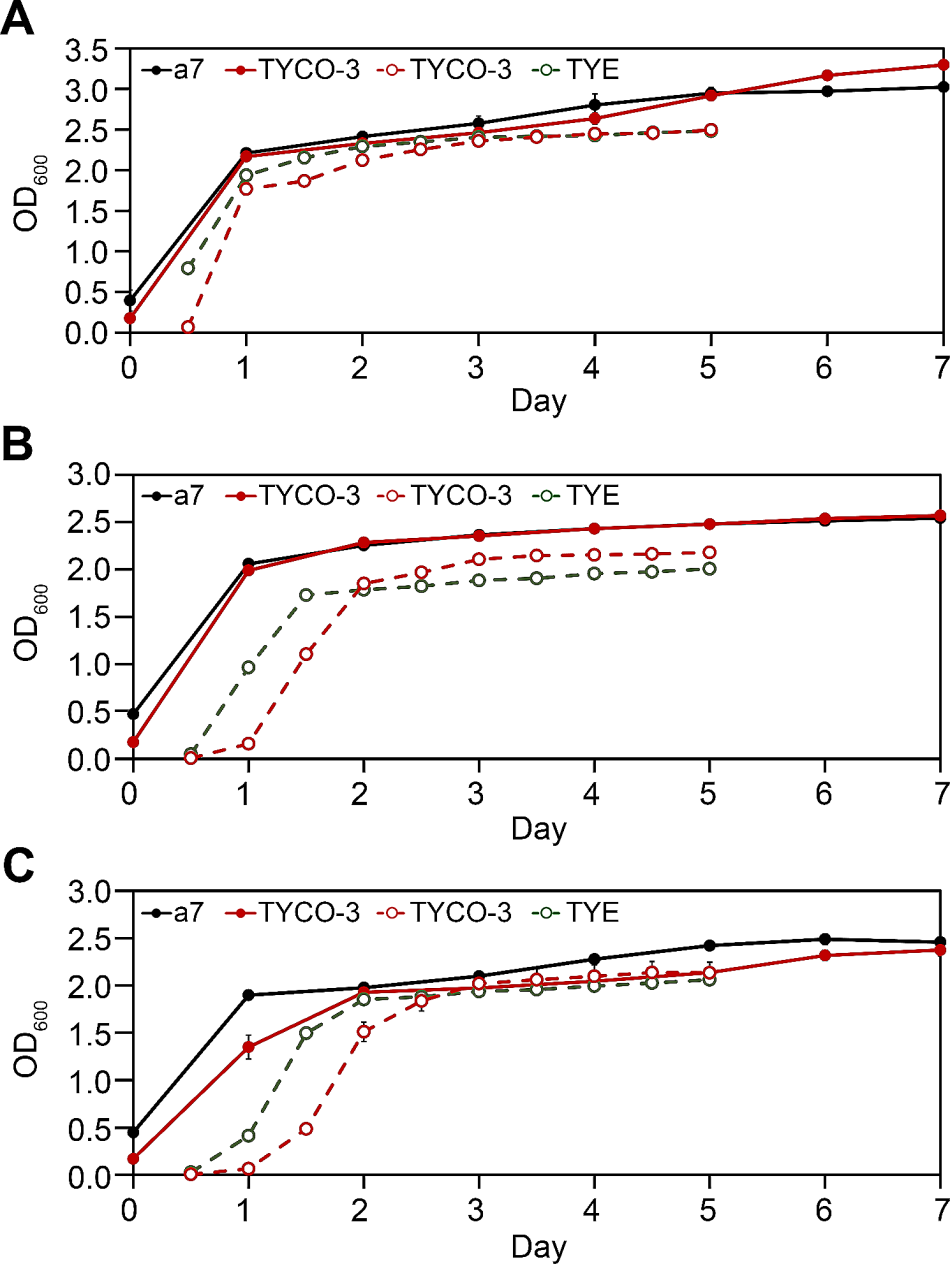
Growth curves of co-transformants at 30 °C (**A**), 25 °C (**B**), and 20 °C (**C**). Solid lines are growth curves in YEPD broth while dashed lines are those in the *ura*^−^ selective broth. Strains are recipient *S. cerevisiae* 2805-a7, mock transformant TYE, and co-transformant TYCO-3. Y and X axis respectively represent the value of optical density at 600 nm (OD_600_) and days after cultivation

The cell growth of co-transformants, a mock transformant, and a recipient *S. cerevisiae* 2805-a7 strain were compared in our culture condition (Fig. 5). When cells were grown in YEPD broth at 30 °C all strains showed similar growth curves; exponential growth until 1 day, then retarded growing phase till day 6, and thereafter a stationary phase. However, when cells were grown in *ura*^−^ selective broth at 30 °C, significantly less cell growth of the co-transformant than the mock transformant was observed during the exponential growth phase. The impaired cell growth of co-transformant confirmed that the expression of the *Cppks1* gene for phleichrome biosynthesis severely affected cell growth. Thus, in *ura*^−^ media, selection pressure forced the cell to maintain the pYEGPD-Cppks1 vector but the cell growth of the co-transformant was negatively affected due to the expression of the accompanying *Cppks1* gene within the pYEGPD-Cppks1 vector. Considering the growth of mock transformants, this is not because of the simple metabolic burden by the presence of episomal vector, but rather is a result of the biosynthesis of a metabolite such as phleichrome with antimicrobial activity.

Similar temporal growth patterns including exponential, early stationary, and stationary phases were observed at both 25 °C and 20 °C as those at 30 °C. The maximum density of cell growth, however, decreased as the temperature fell. Interestingly, while the maximum OD_600_ showed remarkable changes depending on temperatures (OD_600_ of > 3.0 at 30 °C to OD_600_ of < 2.5 at 20 °C) in YEPD broth, relatively small changes, if any, (OD_600_ of ∼ 1.7) were observed in the *ura*^−^ selective media.

No sign of having lost the integrative plasmid pYIGPD-npgA were observed throughout our experiment.

### Effect of culture conditions for phleichrome production

**Fig. 6:**
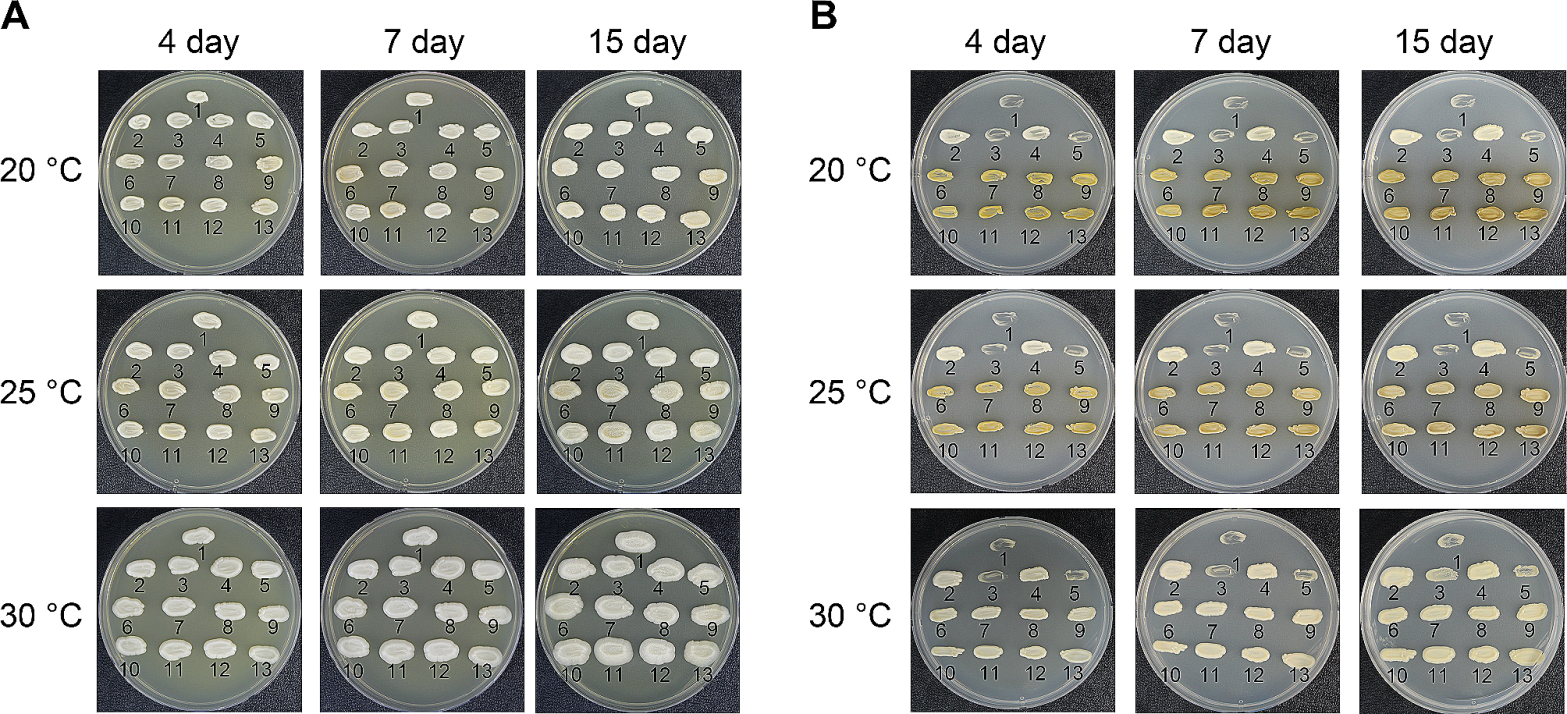
Colony morphology of the selected co-transformants cultured on nonselective YEPD (**A**) and selective *ura*^*−*^ (**B**) media. Culture period and incubating temperatures are indicated at the top and on the left, respectively. Numbers at the bottom of the colony represent the strains: 1; *S. cerevisiae* 2805-a7, 2; TYE (a mock transformant using pYEGPD-TER vector), 3; TYI (a mock transformant using pYEIGPD-TER vector), 4; TYECPPKS1 (a transformant with only a *Cppks1* gene), 5; TYINPGA (a transformant with only a *npgA* gene), 6–13; co-transformant (TYCO)-1, 2, 3, 4, 5, 6, 7, and 8

Because phleichrome possesses a characteristic deep red pigment in the mycelia and culture medium, we looked for changes in the colour of colonies grown under standard culture conditions. As shown in Fig. 6A, no consistently discernible changes in colour were observed on YEPD nonselective solid media. Since selection pressure is required to maintain the high level of the recombinant episomal vector and our recent study demonstrated superior expression of the target gene at lower temperatures [44], we tested our co-transformants on selective media and lowered temperatures (20 °C and 25 °C). When co-transformants were streaked on *ura*^*−*^ selective plate at 20 °C and 25 °C, colour changes in the co-transformants colonies began to appear after the colony morphology of streaked cells showed the fully-grown colony shape (usually after more than four days of incubation) on a *ura*^*−*^ selective plate (Fig. 6B). Interestingly, no discernable color change was observed at 30 °C after a corresponding incubation time (Fig. 6). No changes were observed on the nonselective YEPD plate regardless of strains and temperatures. The changed color was not anticipated pinkish hue, but rather a diffused brown. As expected, no changes were observed from cultures of recipient host *S. cerevisiae* 2805-a7 strain, mock-transformants, and transformants using only either *Cppks1* or *npgA* gene.

**Fig. 7:**
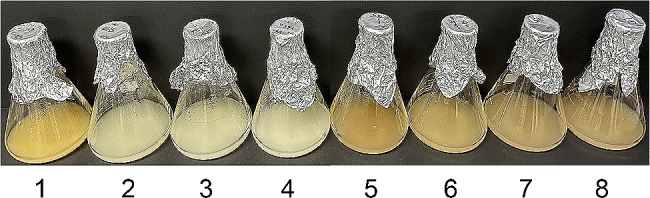
Culture broth of selected co-transformants cultured in selective *ura*^*-*^ liquid media. Flasks were cultured for 10 days at 20 °C. Numbers at the bottom of flasks represent the strains: 1; *S. cerevisiae* 2805-a7 cultured in YEPD medium, 2; TYECPPKS1 (a transformant with only a *Cppks1* gene), 3; TYE (a mock transformant using pYEGPD-TER vector), 4; TYINPGA (a transformant with only a *npgA* gene) cultured in *leu*^*-*^ medium, 5–8; co-transformant (TYCO)-1, 3, 7, and 8. Note that the brownish color of *S. cerevisiae* 2805-a7 reflects the color of the YEPD broth

Changes in colour of colonies were also examined using liquid cultures. Colour changes were observed when co-transformants were incubated in a selective broth at 20 °C and 25 °C, with no changes observed in other culture conditions (YEPD or 30 °C) (Fig. 7), a result consistent with those of the culture plate. Since the colour changes were specific to the co-transformants, we concluded that these colour changes occurred because of our genetic manipulation. Therefore, we examined the nature of the colour changes, i.e., the presence of phleichrome, in these selected co-transformants grown on the *ura*^*−*^ selective media at lowered temperatures of 20 °C and 25 °C using TLC and LC/MS/MS. In addition, considering the plasmid instability and detrimental effect of phleichrome on cell growth, a regulatable promoter instead of the constitutive GPD promoter used in this study may resolve instability and productivity problems.

### TLC analysis of the presence of phleichrome

**Fig. 8:**
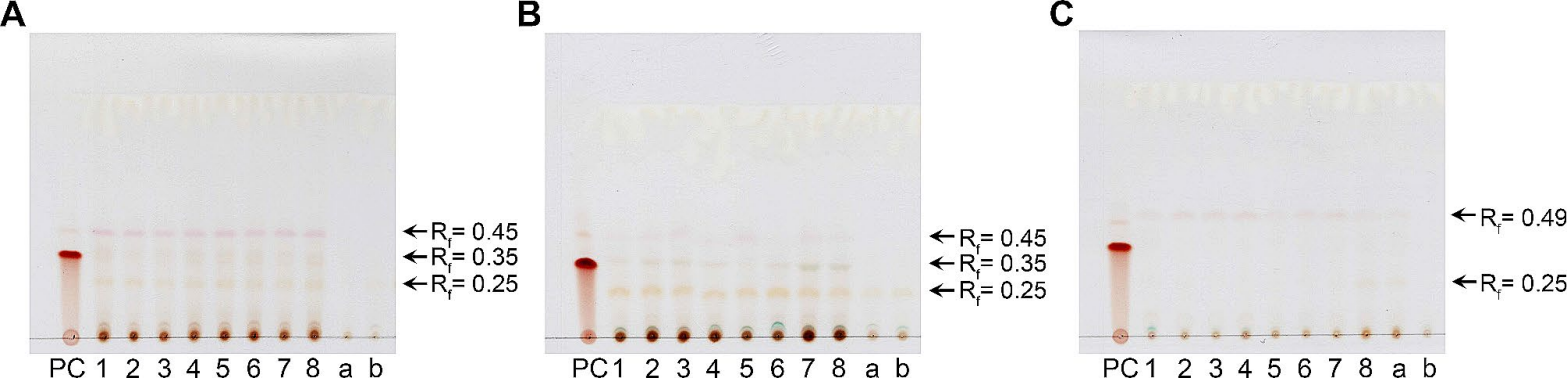
TLC analysis of EtOAc-extracted pigments from cultures grown at 20 ℃ (**A**), 25 ℃ (**B**), and 30 ℃ (**C**). PC represents purified phleichrome. Lanes 1–8 are sample preparation from co-tansformants (TYCO-1, 2, 3, 4, 5, 6, 7, and 8). Lanes a and b contain samples from transformant with only a *Cppks1* gene (TYECPPKS1) and mock transformant (TYE). Note that TYE at 30 ℃ has a faint but discernable band

We performed a TLC analysis using an EtOAc extract of the co-transformant (Fig. 8). EtOAc extract of co-transformants cultured in the *ura*^*−*^ selective liquid media at 20 °C and 25 °C revealed the presence of brownish bands with Rf values around 0.45 and 0.35, which were similar to those of the purified phleichrome from *C. phlei* representing phleichrome and its derivative (Fig. 8A and B). These TLC bands were specific to the co-transformants cultured in the *ura*^*−*^ selective media at the lowered temperatures of 20 °C and 25 °C. No other EtOAc extracts from the recipient *S. cerevisiae* 2805-a7 strain and other control strains showed these corresponding spots. Co-transformants grown in nonselective YEPD or at 30 °C did not show these spots either. In addition, only the EtOAc extract from the cells showing the colour change had these additional bands; even in the EtOAc extract of the co-transformants cultured in the *ura*^*−*^ selective media at 25 °C it was hard to detect these additional bands if they showed no color change yet due to the insufficient culture period. These results indicated that the color change caused by putative pigmentation is attributable to these TLC bands. The brownish TLC bands were thereafter independently scraped off, extracted with methanol, and assessed for the presence of phleichrome by LC/MS/MS.

In addition to those pigments responsible for the change in color of the colony, there was an additional band at a lower Rf value around 0.25, though that was not nearly as strong as those of upper two bands and not consistent throughout all of the TLC analysis (Fig. 8A and B). This additional band, which was observed in the EtOAc extracts of all strains cultured at lower temperatures, suggested that although not as distinctive as those of engineered pigments with higher Rf values, there was an indigenous yeast-produced other pigment that could be extracted by EtOAc treatment.

**Fig. 9:**
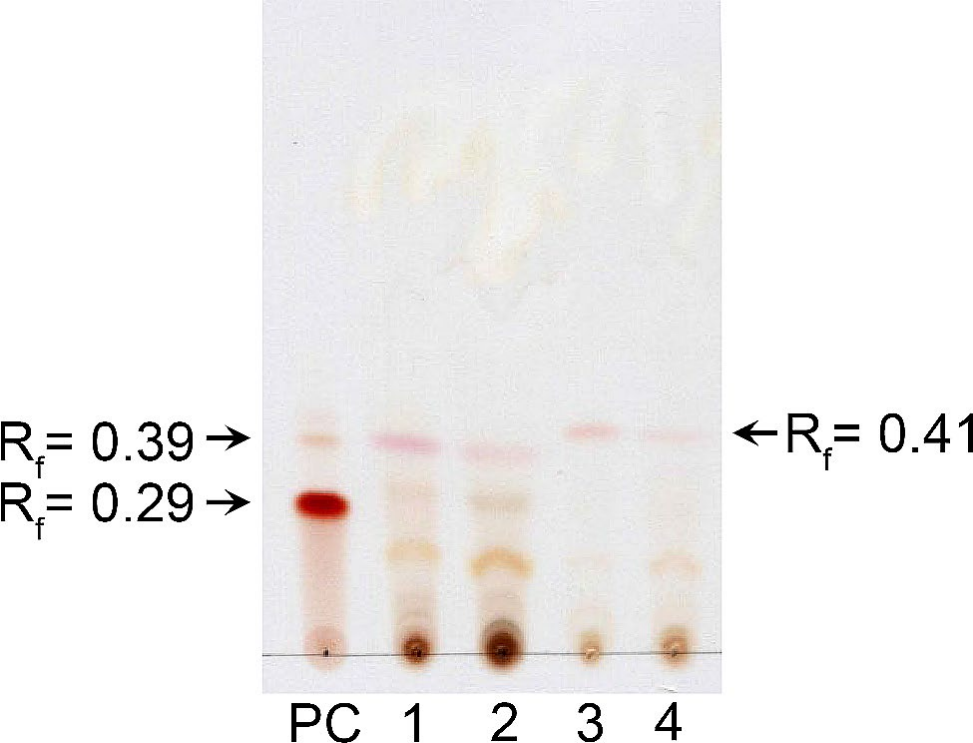
TLC of EtOAc-extracted pigments. Lanes 1–3 contain samples of EtOAc-extract from a co-transformant (TYCO-1) cultured at 20 ℃, 25 ℃, and 30 ℃. Lane 4 contains a sample from a transformant with only a *Cppks1* gene (TYECPPKS1). PC represents purified phleichrome

Interestingly, EtOAc extracts of the co-transformants and other control strains grown at 30 °C showed a pigment band with an Rf value of 0.49, which differed from those of control phleichrome and co-transformants cultured at lower temperature (Fig. 8C). In addition, when EtOAc extracts of the co-transformants cultured at lower temperature and control phleichrome were analysed on the same TLC plate with EtOAc extract at 30 °C, their Rf values differed from each other (Fig. 9). Yeast strains were able to produce another indigenous pigment at 30 °C that was different from the engineered pigment. In addition, changing the temperature from lower levels to 30 °C repressed the biosynthetic pathway of phleichrome at lower temperatures but induced the totally different biosynthetic pathway for an additional pigment. Thus, our results strongly suggest that alterations to the temperature were responsible for profound biosynthetic metabolic changes, as opposed to a simple slow-down of the on-going metabolic processes.

### LC/MS/MS analysis of the pigment from co-transformants

**Fig. 10:**
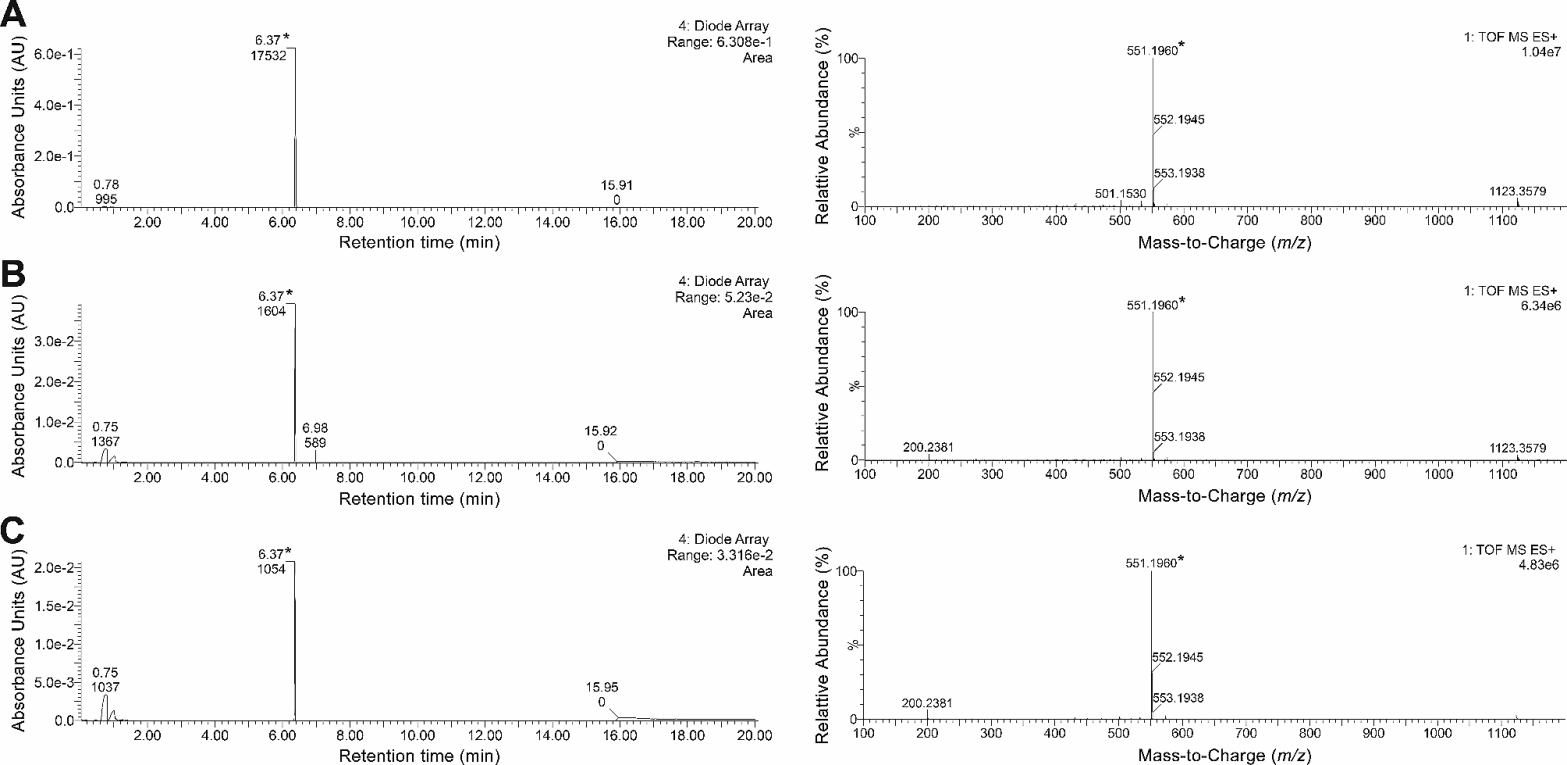
LC/MS/MS of pigments extracted from co-transformants. Samples were purified phleichrome pigment **(A)** corresponding to an upper spot **(B)** and a lower spot **(C)** from co-transformant TYCO-7. Chromatogram of HPLC followed by HRMS analysis with extended MS ranges is shown in the left and right panels, respectively. The star indicates a peak showing the same molecular weight as the pure control phleichrome

As shown in Fig. 10, LC/MS/MS revealed that methanol extract of TLC spots from the co-transformants cultured at 20 °C and 25 °C had peaks with the same retention time and molecular weight (MW 551.1960) as the control purified phleichrome. Phleichrome was detected from both TLC bands with Rf values of 0.35 and 0.45, which were specific to the co-transformants. However, no discernable peak with the same molecular weight of phleichrome was found from methanol extract of the TLC spot from the mock transformant cultured at 25 °C. Nor did the distinct band with a higher Rf value of 0.49 from the EtOAc extract at 30 °C show the presence of phleichrome. Our TLC analysis showing the presence of phleichrome from both distinctive bands of Rf values of 0.35 and 0.45 revealed that the engineered phleichrome existed as a structural isomer with a different polarity. This was also observed in the purified phleichrome that showed two distinct TLC bands. Based on the intensity of the TLC bands and LC/MS/MS peak area, the estimated yield of phleichrome was 0.79 mg/L of yeast culture. Our LC/MS/MS analysis of the engineered phleichrome with an identical molecular mass revealed similar (not always identical) Rf values to the reference phleichrome suggesting that the engineered phleichrome possesses a slightly different polarity or very closely related intermediates. Successful heterologous expression of fungal perylenequinones using yeast [24–26] and the presence of *O*-methyltransferase in the yeast genome database (https://yeast.biocyc.org/) strongly suggested that the presence of *O*-methylation of the nor-toralactone, a common aromatic polyketide precursor synthesized by the PKS gene, followed by oxidative coupling in yeast, biosynthesized the common pentacyclic core of phleichrome. However, our sequence analysis of a fosmid clone of *Cppks1* revealed no synteny with the recently identified gene clusters for perylenequinone biosynthesis [10, 45, 46]. Therefore, this heterogeneity is likely associated with the different genetic backgrounds of the host cells (*S. cerevisiae* vs. *C. phlei*) or perhaps simple differences in the purity of the engineered phleichrome in the dissolving solution. Further studies are required to characterize the enzymatic activities for the process of nor-toralactone undergoes in *S. cerevisiae* and to identify whether products other than phleichrome or biosynthetic pathway intermediates are produced.

## Conclusion

Our results demonstrate that *S. cerevisiae* can be metabolically engineered via co-transformation with two genes of *npgA* and *Cppks1* to produce phleichrome. Plasmid instability and growth inhibition caused by the engineered phleichrome suggest that the synthetic phleichrome maintains phleichrome’s biological functions. As colour changes occurred only under certain growth conditions, such as low temperature and with selective media, *de novo* synthesis and accumulation of the corresponding phleichrome must result from a complex interplay of factors. The highest phleichrome yield from the selected transformant was 0.79 mg/L of cells cultured in *ura*^−^ selective broth for 10 days at 20 °C.

Our research demonstrates a novel method for producing fungal pigments, such as phleichrome. Numerous fungal pigments are synthesized by PKS genes, and more PKS genes are awaiting characterization. However, fungi have multiple PKS genes in their genome, many of which are functionally redundant, rendering it difficult to precisely define the characteristics of each PKS gene, including which PKS gene is responsible for which pigment. Ultimately, our engineered system to produce fungal pigments can be applied to discover the direct relationship between various PKS genes and their corresponding metabolic products.

## Data Availability

No datasets were generated or analysed during the current study.

## Abbreviations

NR-PKs: non-reducing polyketide synthase
PPTase: phosphopantetheinyl transferase
TLC: thin layer chromatography
PDT: photodynamic therapy
PKS: Polyketide synthase
6-MSA: 6-methylsalicylic acid
ACP: acyl carrier protein
GPD: glyceraldehyde-3-phosphate dehydrogenase
GAL7: galactose-1-phosphate uridyl transferase
EtOAc: ethyl acetate
CFUs: colony forming units
*ura*^-^: uracil-deficient
*leu*^-^: leucine-deficient
